# Identification of *Pseudomonas aeruginosa* From the Skin Ulcer Disease of Crocodile Lizards (*Shinisaurus crocodilurus*) and Probiotics as the Control Measure

**DOI:** 10.3389/fvets.2022.850684

**Published:** 2022-04-21

**Authors:** Yi Xiong, Qiong Wu, Xudong Qin, Chengsheng Yang, Shuyi Luo, Jiasong He, Qingzhen Cheng, Zhengjun Wu

**Affiliations:** ^1^Key Laboratory of Ecology of Rare and Endangered Species and Environmental Protection, Ministry of Education, Guilin, China; ^2^Guangxi Key Laboratory of Rare and Endangered Animal Ecology, College of Life Science, Guangxi Normal University, Guilin, China; ^3^Daguishan National Nature Reserve for Crocodile Lizards, Hezhou, China

**Keywords:** *Pseudomonas aeruginosa*, *Shinisaurus crocodilurus*, pathogenicity (infectivity), probiotics, animal conservation

## Abstract

The crocodile lizard (*Shinisaurus crocodilurus*) is an endangered ancient reptile species. Captive breeding is an important conservation measure for the potential restoration and recovery of their wild populations. However, a skin ulcer disease caused by an unknown pathogen has become a serious threat to captive breeding individuals. In the current study, based on microbial isolation, we identified *Pseudomonas aeruginosa* as the dominant pathogen in skin ulcer disease. Chinese skinks (*Plestiodon chinensis*) were used to verify the pathogenicity of *P. aeruginosa* in skin ulcer disease *in vivo*. As expected, subcutaneous inoculation of *P. aeruginosa* induced skin disease in healthy skinks and *P. aeruginosa* was re-isolated from the induced skin ulcers. Therefore, *P. aeruginosa*, an opportunistic and ubiquitous pathogen that causes a wide range of infections, appears to be the main pathogen of the skin disease affecting crocodile lizards. In the aquaculture industry, probiotics are widely used in the prevention and control of animal diseases caused by such pathogens. Here, we administered probiotics to the breeding crocodile lizards for 6 months. The three experiment groups treated with different kinds of probiotics showed significance at controlling case incidence. Three of the four groups treated with probiotics showed significant disease prevention (Effective Microorganisms mixed probiotics *P* = 0.0374; Double-dose Effective Microorganisms, *P* = 0.0299; *Bacillus subtilis, P* = 0.0140, *T*-test), and CFUs in the water of the breeding enclosures were also inhibited after probiotics usage (*P* < 0.001, *T*-test). Our study demonstrated the role of *Pseudomonas aeruginosa* in development of skin ulcer disease of crocodile lizards in a local zoo and offered the probiotic-based method for control measurements, which would be of benefit for the conservation of endangered reptiles.

## Introduction

While artificial breeding holds promise as a method for protecting endangered species, its successful application remains limited (1–3). One reason for previous failures is that artificial populations, including reptiles, are often housed under unnatural high-density conditions. For example, in Gandong Station at Daguishan National Nature Reserve, captive lizards were previously housed in enclosures at a density of 2–3 individuals per m^2^, which may increase the risk of fights and injury and thus susceptibility to various diseases and infections (2, 4–9). Several recent studies have shown that infection in breeding reptiles can impact protection efforts and health endeavors (4, 6, 7, 10–12). Thus, infections and diseases in captive populations need to be considered not only to ensure that reserves do not squander their limited resources but to ensure the best chance of successful breeding. However, limited by various protection laws, lack of funds, and shortage of professionals, protection work in this field remains poor.

The crocodile lizard (*Shinisaurus crocodilurus*) is a highly endangered reptile found in the streams and forests (800–1,200 m altitude) of the Guangxi and Guangdong Provinces in China and in North Vietnam. The crocodile lizard is currently classified as a first-class protected animal in China and is listed in the Red List of Threatened Species of the IUCN and in CITES Appendix I. At present, only 1,200 lizards are thought to remain in China, with a small population of 150 in Vietnam (3, 13). All populations in China are small and many are isolated due to habitat fragmentation. Thus, to recover these wild populations, artificial populations have been established for captive breeding and eventual release. However, several diseases have been reported in these captive populations, including an obvious skin disease. This disease manifests as ulcers and swellings around the limbs, and cases of death always present with intestinal inflammation, indicating severe bacterial infection. According to the records of the breeding stations at the Daguishan and Luokeng nature reserves, this disease kills over 30% of infected juveniles (aged <1 year). In addition to the high mortality rate, the disease also causes stunted growth, further undermining attempts to recover wild population. Of concern, the release of these captive-bred lizards may introduce the disease to unprotected wild populations, further impacting restoration efforts and population decline (2, 14–16). Several reports also suggest that humans are at risk of zoonotic infections from reptiles (6, 8, 17–19). Thus, prevention and control of this disease is a critical challenge. Jiang et al. (14) used metataxonomics to identify the pathogens related to cutaneous granuloma disease in crocodile lizards and Omondi David et al. (20) also use molecular method to detect the pathogen in reptiles (14, 16, 20). However, identification of pathogens in animal skin diseases remains rare. Thus in our earlier study (21), we explored the epidemiology of this skin disease and identified several potential pathogens by sequencing. Notably, we determined the incidence rate and various symptoms of the disease and used 16S rRNA gene sequencing to detect wound microbiota in 12 diseased individuals, with *Pseudomonas* identified as the most likely pathogen.

Among other reptiles, researches mainly focused on larger animals like turtles or snakes. As the spreading of COVID-19, people are more and more aware of the wild or semi-wild breeding animals for any possible pathogens, infection cases among reptiles are reported more frequently these years, such as Salmonella infection, Nematoda infections and Nidovirus infections (22–29). Among these reports, *Pseudomonas* infections among lizards are reported since decades before. Lizards were also highlighted because reptiles (pets) were assumed to transfer pathogens to human recent years (30–32). The internal environment for these pathogens, microbiota, both in guts microbiota and skin microbiota, were assumed to play important roles in disease occurrence (33). Microbiota in the gut and skin can fight against pathogens and enhance body immunity, with many studies identifying a relationship between probiotics and health (34–36). Probiotics have been used in livestock and fish breeding, as well as in several human diseases (such as mild enteritis, acne, and atopic dermatitis) (37, 38). With increasing antibiotic resistance, probiotics are an environmentally friendly replacement that may ease some of these issues (39, 40). Probiotics are extensively used in livestock breeding and can effectively prevent disease in animals due to defensive surface protection and immune enhancement, and there are some mechanism researches among livestock (such as Domestic chickens)and human (33, 41). In addition, among marine animals, probiotics are widely applied to ameliorate water quality during breeding. Thus, as a water-living reptile, we assumed that probiotics may help reduce disease incidence in crocodile lizards (39, 42). In the current study, we identified related pathogens *in vitro* and *in vivo* and explored the provision of probiotics as an effective measure to prevent skin ulcer disease in these reptiles.

## Methods

### Ethical Statement

All experimental procedures were conducted following the guidelines approved by the Institutional Animal Care and Use Committee (IACUC) of Guangxi Normal University (Reference Number: 202105-003).

### Bacterial Isolation and Cultivation

Based on our former research (21), we assumed that water in the enclosures may be the main medium for disease outbreaks. Thus, we collected water samples from the enclosures for pathogen extraction and identification. According to our former research, *Pseudomonas* was considered the most likely pathogen, so we used *Pseudomonas* selective media, including *Pseudomonas* CFC selective agar and additives (Qingdao Hope Bio-Technology Co., Ltd., Qingdao, China) and glycerol (LIRCON Medical Technology Co., Ltd., Dezhou, China) to isolate and cultivate *Pseudomonas* from five breeding pool water samples. We used colony forming units (CFUs) to count bacterial density in these samples. Bacterial cultivation was conducted at 30°C in an incubator for 48 h (43). After that we had a series of bio-chemical tests for the cultivated colonies, including cultivation on Cetrimide agar plates (Qingdao Hope Bio-Technology Co., Ltd., Qingdao, China), Gram staining, Oxidase test (Oxidase test paper made by Qingdao Hope Bio-Technology Co., Ltd., Qingdao, China), Pyocyanin test (*Pseudomonas* Agar Medium for Detection of Pyocyanin, PDP produced by Qingdao Hope Bio-Technology Co., Ltd., Qingdao, China) and 42°C cultivation test.

After cultivation, colony samples were collected and DNA was extracted (44). The 16S rRNA gene was amplified using 338F (5'-GTGCCAGCMGCCGCGGTAA-3') and 806R (5'-GGACTACHVGGGTWTCTAAT-3') primers. Bacteria were annotated by matching similarity results in the NCBI database using BLASTn (https://blast.ncbi.nlm.nih.gov). These results were genetically and morphologically compared to our former cultivation of *P. aeruginosa* standard strain (CMCC[B]10104) (Qingdao Hope Bio-Technology Co., Ltd., Qingdao, China). The original colonies were deposited in agar under 28°C for infection verification.

### Infection Verification

As crocodile lizards are a protected species, we chose closely related Chinese skinks (*Plestiodon chinensis*) for *in vivo* testing and constructed an artificial infection animal model by creating abrasions on the skin of the skinks (14, 16, 45–48). Jiang et al. (14) also used Chinese skinks as an animal model for cutaneous granuloma identification in crocodile lizards. Here, all experimental skinks were adult males obtained from the Longmeng Lizard Breeding Station (Business license: 102620002940) in China. A scalpel was first used to make a small round wound (0.5 cm in diameter) under sterile conditions, after which *P. aeruginosa* fluid (1 ml, 1CFU:10^5^ ml in solution) was administered to the wounds (14, 16, 33, 41). Two control groups were established, including a no-operation group and a wound-only group. Two separate wounds were created on each of the eight skinks (14, 16).

The skinks were fed crickets once every 2 days and housed under a high humidity (>85%) environment, the same as the crocodile lizard breeding pools (3, 13), with limited bacterial exposure. As soon as the wounds showed obvious infection, we compared them to the crocodile lizard wounds in appearance. The skinks were then sacrificed *via* oral administration of formalin (30 ml). Tissue sections around the wounds were collected for hematoxylin and eosin (H&E) staining (49) and microscopic observation.

### Disease Prevention

Since the Ministry of Agriculture and Rural Affairs of the People's Republic of China (http://www.moa.gov.cn/) announced Proclamation 194 in January 2020 restricting the use of antibiotics in China, other effective methods must be used to prevent animal disease. Here, we evaluated the effects of probiotics on skin disease prevention in crocodile lizards at the captive facility. We recorded all infection cases in 2021 (with probiotic use) and compared the statistics to those recorded in 2019 (with no probiotic use). The disease incidence rate of each group divided by the incidence rate of the control group was determined as an indicator of the effectiveness of the probiotics.

Starting in April 2021, we used three kinds of probiotics and four crocodile lizard groups at the Gandong breeding station to test the effects on skin disease incidence (39, 40, 42, 50–53). Each group contained about 40 animals, according to the maximum allowed by the management regulations of Daguishan National Nature Reserve. The experiment lasted 6 months, from April to October (non-hibernation period).

The four treatment groups were provided with probiotic supplements in food as well as external application. The groups included Group A [30 mg/kg effective microorganism (EM) mixed probiotics, produced by Morishita Jintan Co., Ltd., Japan, containing ~80 probiotics, e.g., *Bacillus subtilis, Saccharomyces cerevisiae, Lactobacillus, Cyanobacteria*], Group B (60 mg/kg EM mixed probiotics, produced by Morishita Jintan Co., Ltd., Japan, containing ~80 probiotics, e.g., *Bacillus subtilis, Saccharomyces cerevisiae, Lactobacillus*, and *Cyanobacteria*), Group C (30 mg/kg of *Bacillus subtilis*, produced by Qiang Xing Biotech Corporation, Beihai, China), and Group D (30 mg/kg of *Saccharomyces cerevisiae*, produced by Angel Yeast Corporation, Yichang, China). All groups were divided under systematic sampling. Groups B, C, and D contained equal enclosures of different age and sex (e.g., one adult male, one adult female, two juveniles, and two sub-adult enclosures). Group A contained the remaining lizards not contained in Groups B, C, and D. The doses used in Groups A, C, and D followed the recommendations of the manufacturer, while the dose used in Group B was doubled. All treatment groups were fed probiotics once every 2 days (same as food supply), and water in the enclosures was treated once per week by putting probiotics solutions into pools.

We recorded the incidence rate and death rate in all groups from May to October 2021, as well as disease statistics from the no-probiotics control group (39, 40, 42, 50–53). These data were used for comparative and effectiveness analyses. All crocodile lizards were provided with the same food, including crickets, earthworms, and mealworms. Chi-square test of total incidence cases, one-way analysis of variance (ANOVA) between all groups, and *T-*test between each two groups were applied to test the effectiveness of the probiotics, with *P*-values recorded. Statistical analyses were performed using GraphPad Prism (v8.4.2).

### Microbiota Test

In October 2021, we performed skin microbiota 16S rRNA gene high-throughput sequencing to determine bacterial infection after probiotic treatment. We used the Levine cotton swab method (54) to collect skin microbiota samples from crocodile lizards from each group. For sequencing, we constructed a 20-μl reaction system using TransGen AP221-02: TranStart Fastpfu Polymerase (TransGen Biotech, Beijing, China), including 5 × FastPfu Buffer (4 μl), 2.5 mM dNTPs (2 μl), forward primer (5 μM, 0.8 μl), reverse primer (5 μM, 0.8 μl), FastPfu Polymerase (0.4 μl), bovine serum albumin (BSA, 0.2 μl), template DNA (10 ng), and distilled water (to volume). Primers 338F (5'-GTGCCAGCMGCCGCGGTAA-3') and 806R (5'-GGACTACHVGGGTWTCTAAT-3') were used to amplify the V3 and V4 hypervariable regions of the 16S rRNA gene. The amplicon library was prepared using a TruSeq R DNA PCR-Free Sample Preparation Kit (Illumina Inc., USA). Sequencing on the Illumina MiSeq platform (150-bp paired-end reads) was performed by the Majorbio Corporation (Shanghai, China). After sequencing, the operational taxonomic units (OTUs) were constructed, and raw tags were filtered using the QIIME2 package (v2020.8) to remove low-quality and chimeric sequences. Sequences with ≥97% similarity were assigned to the same OTU using Uparse in USEARCH (v10). A representative sequence for each OTU was annotated using Mothur1 by searching the SILVA database (https://www.arb-silva.de/) (threshold = 0.8). OTU abundance of *P. aeruginosa* and skin microbiota composition were compared to groups and 16S rRNA gene analysis results in our previous study (21). All raw sequences obtained from high-throughput sequencing were deposited in the NCBI Sequence Read Archive (SRA) under accession number PRJNA789133.

We sampled water in five random enclosures in Group A in August 2021 to assess the effectiveness of water purification (Groups B, C, and D had so few enclosures they were not included). Total CFUs and *P. aeruginosa* CFUs were recorded. Statistical analyses (*T*-tests) were performed using GraphPad Prism (v8.4.2).

## Results

### Pathogen Verification

**Figure 1:**
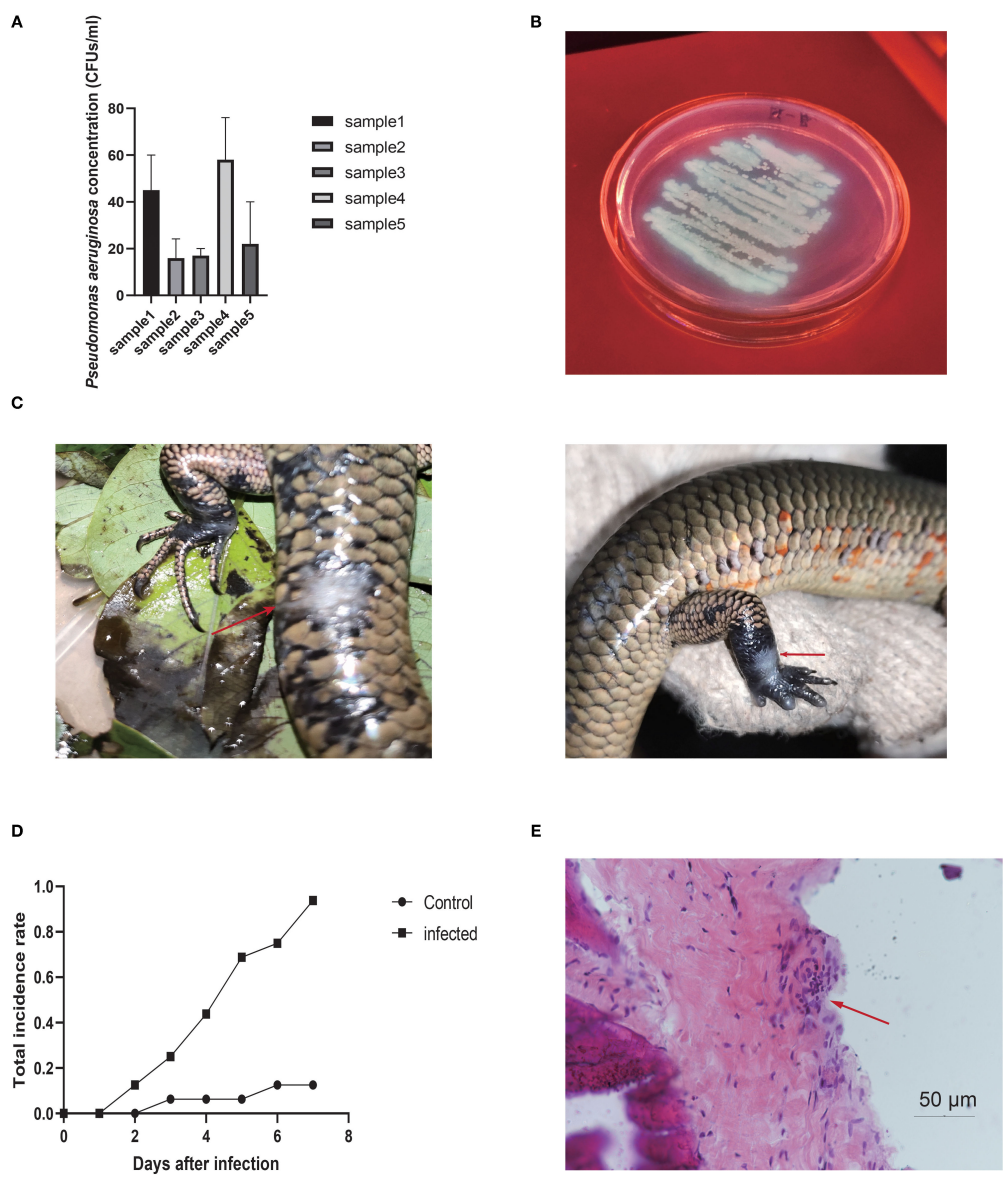
**(A)** *Pseudomonas aeruginosa* CFUs isolated from five groups of water samples in breeding enclosures. **(B)** Colonies of *P. aeruginosa* isolated from water samples on agar plates. **(C)** *Pseudomonas aeruginosa*-infected wounds in Chinese skinks. Arrows point to ulcers. **(D)** Infection rate under *P. aeruginosa* strain compared to control group, with obvious differences between groups. **(E)** *Pseudomonas aeruginosa*-infected wound under the microscope. Arrows point to inflammatory changes.

Tissue samples from ulcerated and healthy crocodile lizard skin were obtained using Levine cotton swabs. The samples were then cultured in selective *P. aeruginosa* medium. We found typical colonies with green water-soluble colorants in the ulcer skin samples, but no colonies in the healthy skin samples (Figure 1B). Moreover, we found *P. aeruginosa* proliferation in the breeding pools at ~40 CFUs/ml according to five water samples (Figure 1A), suggesting that *P. aeruginosa* proliferation occurred during July and August 2021.The biochemical test shows support for *P. aeruginosa* identification (images of tests in Supplementary Material, gram negative, 42°C cultivation positive, Cetrimide agar proliferation positive, Pyocyanin test positive and Oxidase test positive), and all bacterial sequence identities showed >97% similarity to the *P. aeruginosa* standard strain [CMCC(B)10104] (0.98, 0.99, 0.98, 0.97, 0.97, 0.97, 0.98, and 0.98). The green metal-like colonies (as in Figure 1B) supported our assumption morphologically.

*Pseudomonas aeruginosa* infection also caused skin ulcer disease in the Chinese skinks, with white and blue swollen ulcers appearing on the skin around the artificially created wounds, consistent with skin ulcers in the crocodile lizards (Figure 1C). The images showed inflammatory cell infiltration in the interstitial tissues, signifying that true inflammation changes had occurred under the ulcers in Chinese skinks. The wound infection rate reached ~95% 1 week after infection, whereas the wound-only and no-operation groups showed infections rates of 15% and 0%, respectively (Figure 1D). Tissue observation showed inflammatory infiltration in the tissue under the skin (Figure 1E), indicating that *P. aeruginosa* had caused an infection. These results indicate that the existence of open wounds provides the opportunity for disease, but *P. aeruginosa* is the decisive factor.

### Disease Prevention Result

**Figure 2:**
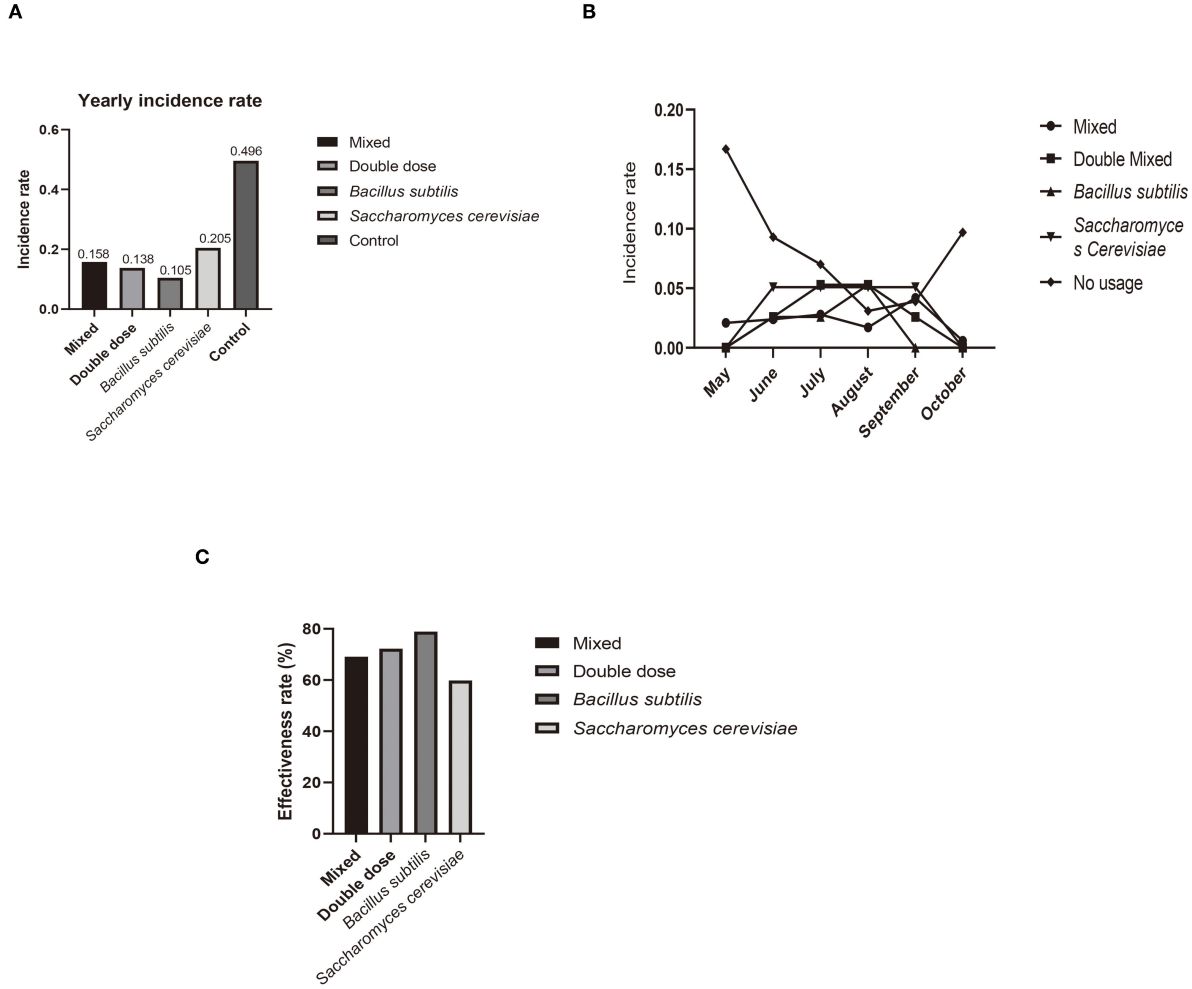
**(A)** Yearly incidence rate of skin disease compared to all four experiment groups and control. **(B)** Incidence rate each month, with peak in May and October not observed in all experimental groups. **(C)** Effectiveness of treatment in each experimental group (compared to control). Former three groups showed a high level of effectiveness groups.

The incidence rate of disease recorded in showed lower level in all four groups. All groups showed significant differences compared to the control group, indicating that probiotic treatment helped prevent disease against *P. aeruginosa* infection (Figure 2A). Notably, the total incidence rate was 49.6% (128/258) in the control group and only 10.3% (40/389) in the experimental groups, with significant differences between the control (no-use) and all experiment groups (total) (χ^2^ = 124.82, *P* < 0.01, chi-square test). The Group A (mixed probiotics) and Group B (double dose) results indicated that increasing the effective concentration of the probiotics dose had little effect on disease prevention, while Group C (*Bacillus subtilis*) exhibited a slight increase in effectiveness among the groups, and one-way ANOVA (GraphPad Prism v8.4.2) showed no significant differences among these experimental groups (*P* = 0.430). The disease incidence rates each month (May to October) indicated that probiotics ameliorated disease outbreak compared with the control, thereby reducing the risk of severe epidemic issues (Figures 2B,C). *T-*test analysis of each group pair showed no significant differences among the experimental groups, but significant differences compared to the control group except Group D (Group A, *P* = 0.0374; Group B, *P* = 0.0299; Group C, *P* = 0.0140; Group D, *P* = 0.0581) (Table 1).

**Table 1:** *T*-test results between groups.

***T*** **-test results between groups (** ***P-*** **value)**					
	**Group A**	**Group B**	**Group C**	**Group D**	**Control group**
Group A	N/A	*P* = 0.6391	*P* = 0.2170	*P* = 0.8772	*P* = 0.0374^*^
Group B	*P* = 0.6391	N/A	*P* = 0.5148	*P* = 0.6077	*P* = 0.0299^*^
Group C	*P* = 0.2170	*P* = 0.5148	N/A	*P* = 0.2625	*P* = 0.0140^*^
Group D	*P* = 0.8772	*P* = 0.6077	*P* = 0.2625	N/A	*P* = 0.0581
Control group	*P* = 0.0374^*^	*P* = 0.0299^*^	*P* = 0.0140^*^	*P* = 0.0581	N/A

*Group A (EM mixed probiotics, P < 0.05), Group B (double-dose EM, P < 0.05), and Group C (Bacillus subtilis, P < 0.05) showed significant difference to control group. Group D (Saccharomyces cerevisiae) did not shows significance to control group*.

* *means significant results (P < 0.05)*.

### Microbiota Functional Analysis

**Figure 3:**
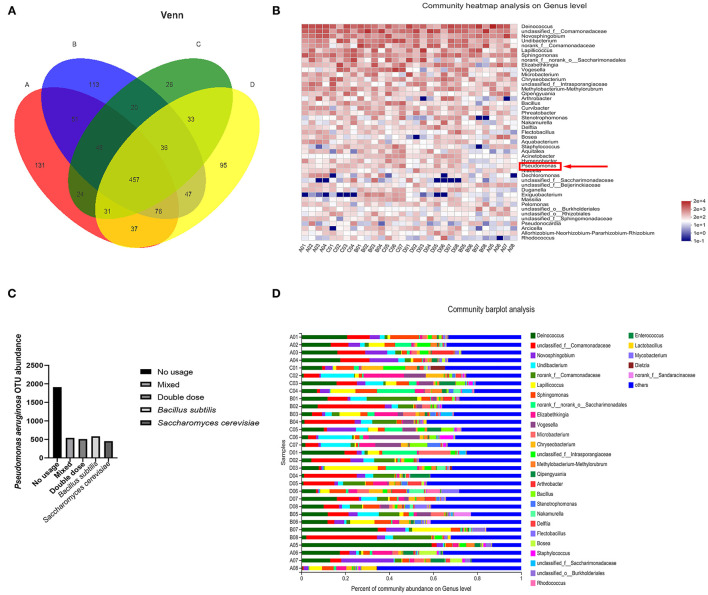
**(A)** Venn diagram of relationship between four experimental groups at genus level from sequencing statistics, with focus on commonality in these groups. **(B)** Heatmap of microbiota in experimental individuals. Pseudomonas (red arrow) is lower in that in control. **(C)** Differences in OTU abundance between experiment and control groups. **(D)** Composition of microbiota in experimental groups. Level of four kinds of pathogen is quite lower than samples from control group (in former research) (19).

After sequencing, we obtained skin microbiota data from 31 samples from all four treatment groups, and those common species in the central part of Venn graph were highlighted (Figure 3A). *P. aeruginosa* levels did not show significant differences among the four groups, but OTU abundance was markedly lower than that in samples from the control group [Figures 3B,C, Supplementary Figure 1 (control group)]. Thus, probiotics had a considerable effect on disease prevention, but there was little difference among treatments in preventing *P. aeruginosa* proliferation. In addition, there was no difference in the effectiveness of doses higher than the minimum effective dose.

The water samples contained 850 CFUs (average) after probiotics usage compared to 37,822 CFUs (average) before probiotics use (*P* < 0.001, *T*-test). At the *P. aeruginosa* level, the samples contained 8.07 CFUs (average) compared to 31.6 CFUs before probiotics usage (*P* < 0.001, *T*-test), suggesting that the use of probiotics can inhibit *P. aeruginosa* proliferation in the water environment of the breeding enclosures.

For microbiota analysis, smooth rare fraction curves showed that sequencing of the skin microbiota was reliable in terms of depth and accuracy (as in Supplementary Figure 2). The dominant genus in the probiotic-treated samples was *Deinococcus* (Figure 3D). *Ralstonia* and *Pseudomonas* were the dominant flora in the untreated crocodile lizard skin, with *Acinetobacter* and *Elizabethkingia* also observed in our former research (21). These four bacteria are all reported as pathogens among humans and animals. Based on sample comparison, the OTUs of the four above mentioned pathogens decreased with statistical significance in the probiotic-treated samples. From these results, the change in skin microbiota was associated with a lower possibility of disease occurrence after probiotics usage.

## Discussion

In the current study, we investigated the pathogenicity of *P. aeruginosa*, with evidence supporting the assumption that *P. aeruginosa* is the pathogen causing crocodile lizard skin infections. Strong evidence supported the source of the *P. aeruginosa* came from water in pools of enclosures. We also examined changes in the disease incidence rate after probiotics use. In general, the probiotics had a positive influence on skin disease prevention. According to the local breeders at Gandong Station, there were many cases of skin disease in the crocodile lizards in 2017 and 2018 (without probiotics usage), although accurate disease rates and lizard numbers were not recorded. However, compared with levels in 2019 (128/258) and 2020 (97/301), the incidence of disease showed a significant and rapid declining trend in 2021 [40/389 (10.3%) from May to October following the introduction of probiotics from 2020].

**Figure 4:**
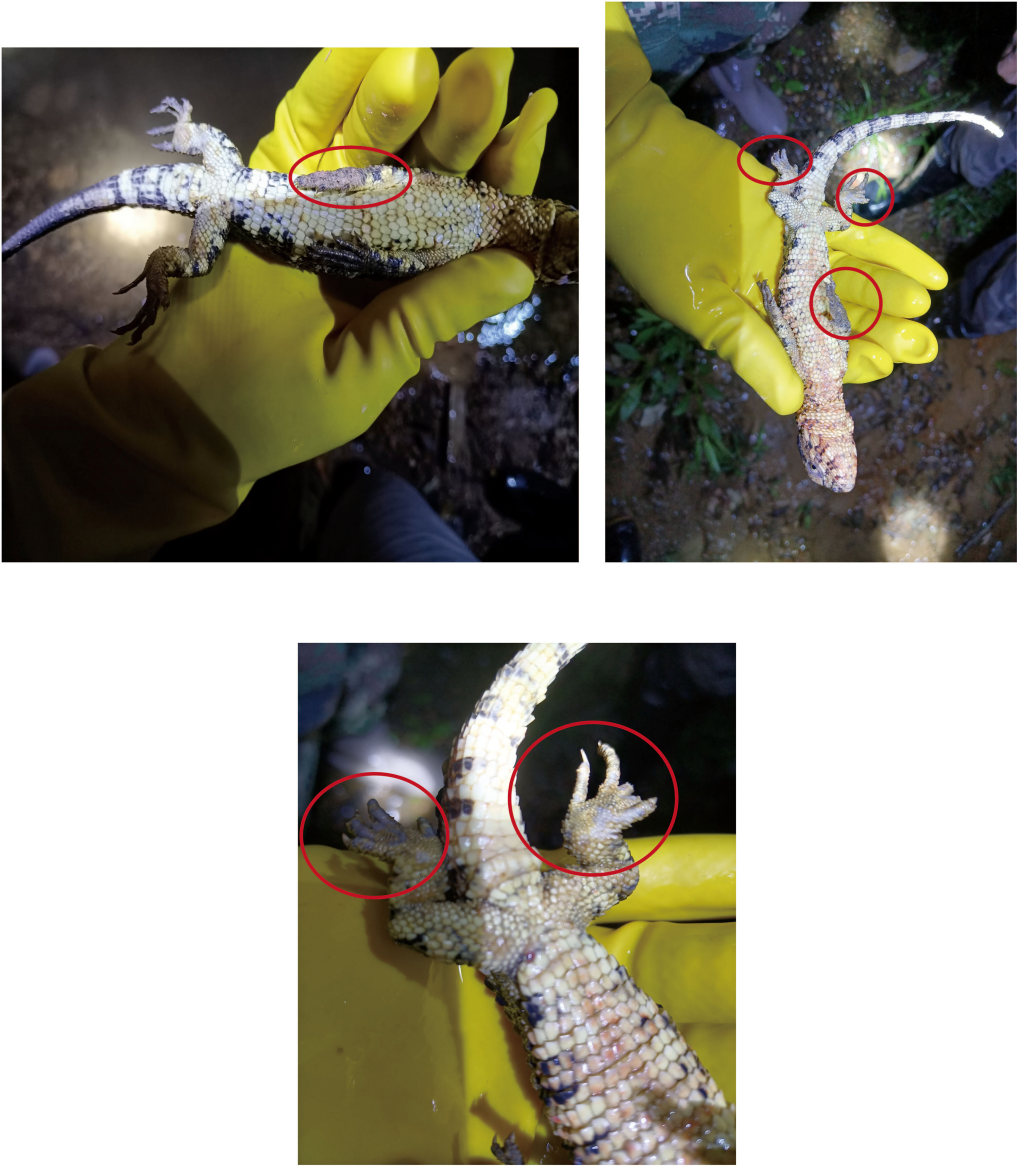
Typical wild case found in 2019 in Yusanchong of Daguishan National Nature Reserve, Hezhou, China. Infection wound is highlighted by a red circle and showed high similarity to captive cases.

Most research on crocodile lizard disease has occurred within the last 5 years. In 2017, *Austwickia chelonae* was reported to cause cutaneous granuloma in crocodile lizards, with pathogenicity also recorded at another crocodile lizard reserve (14). These captive crocodile lizard infections have impacted species protection, with cases now also found in the wild (July 2021, Yusanchong in Daguishan Reserve, Figure 4). However, reports on these diseases in lizards remain limited. Based on our results, crocodile lizard skin disease was highly dependent on the presence of open wounds, and disease lesions were all located on the extremities or on vulnerable areas such as the abdomen. Notably, infection appeared to start from a small skin injury, before progressing to form ulcers and swellings with wound enlargement.

*Pseudomonas aeruginosa* is a well-known opportunistic pathogen causing diverse diseases in humans and animals. There are several reports about the *Pseudomonas aeruginosa* infections among reptiles, stocks and human, mainly cause skin infections and can transfer to septicemia (18, 19, 46, 55). These bacteria can cause skin, respiratory, and digestive system infections and septicemia in reptiles (10–12). In addition, reptiles are considered reservoirs for *P. aeruginosa*, which can spillover and threaten humans and livestock (17–19). Considering the close contact that occurs in zoos and reserves, not only among animals within the same enclosure but also humans and other animals, the spillover risk from crocodile lizards should not be ignored (7, 56). We also observed typical *Saprolegnia ferax* infection symptoms (white branched filaments on wounds) in several dead crocodile lizards, suggesting that co-infection may be possible in severely diseased cases with larger wounds, as supported by the late changes in *Saprolegnia*-infected ulcers after water exposure. Due to the abundance and seriousness of antibiotic resistance in *P. aeruginosa*, as well as the recent prohibition on excessive antibiotic use in China (18, 57, 58), we did not perform an antibiotic sensitivity test (AST). Instead, we studied disease prevention using probiotics rather than antibiotics. Before 2021, captive management at the breeding station relied on 0.1% potassium permanganate solution for body surface disinfection as well as the isolation of cases. In contrast, probiotics provide a cheaper and more environmentally friendly treatment method than disinfectant, without drug-resistance issues (39, 40, 42, 50, 52, 53). Our results showed that disease incidence decreased markedly after probiotic treatment, showing over 60% effectiveness. In the treated crocodile lizards, there was no significant difference in the effectiveness of the different probiotics and no difference in effectiveness at doses above the minimum effective dose. According to our results, the use of probiotics in crocodile lizards should be considered, as supported by previous wound microbial research (33, 43, 59, 60). In addition, as most *P. aeruginosa* exposure occurs in water, water health in the enclosures should also be monitored. At present, the main water supply for the crocodile lizard enclosures comes from nearby streams (belonging to the He River) and improved water treatment could help reduce disease incidence. Reducing population density within the enclosures may also help limit injury and disease. Furthermore, given their wide use in aquacultural fish breeding, probiotics may be a good approach not only for crocodile lizards but also for other protected animals (39, 40, 42, 52, 53). Our results should benefit lizard breeding and lessen the potential of trans-species disease.

However, due to the limitation of pathogenicity research allowed in our laboratory, further experiments on the underlying disease mechanism, including antibiotic resistance tests, are still ongoing. In the current study, we mainly verified the disease and countermeasures at the macro scale. As such, molecular mechanisms of crocodile lizard immunity and pathogens still need to be further studied.

## Data Availability Statement

The datasets presented in this study can be found in online repositories. The names of the repository/repositories and accession number(s) can be found in the article/Supplementary Material.

## Ethics Statement

The animal study was reviewed and approved by Institutional Animal Care and Use Committee (IACUC) of Guangxi Normal University (Reference Number: 202105-003).

## Author Contributions

YX, QW, and ZW designed the research. YX, XQ, SL, JH, and CY participated in the outside experiments. YX and QC participated in the *in vivo* test. YX and QW analyzed the data and wrote the manuscript. ZW revised the manuscript. All authors contributed to the final manuscript.

## Funding

This study was financially supported by the National Natural Science Foundation of China (31760623 and 32160131), Financial Project of State Forestry Administration (V2130211), and Project of Daguishan National Nature Reserve.

## Conflict of Interest

The authors declare that the research was conducted in the absence of any commercial or financial relationships that could be construed as a potential conflict of interest.

## Publisher's Note

All claims expressed in this article are solely those of the authors and do not necessarily represent those of their affiliated organizations, or those of the publisher, the editors and the reviewers. Any product that may be evaluated in this article, or claim that may be made by its manufacturer, is not guaranteed or endorsed by the publisher.

## References

[1] Böhm M Collen B Baillie JE Bowles P Chanson J Cox N. The conservation status of the world's reptiles. Biol Conserv. (2013) 157:372–85. 10.1016/j.biocon.2012.07.015

[2] Harding G Griffiths RA Pavajeau L. Developments in amphibian captive breeding and reintroduction programs. Conserv Biol. (2016) 30:340–9. 10.1111/cobi.12612 26306460

[3] van Schingen M Le MD Ngo HT Pham CT Ha QQ Nguyen TQ. Is there more than one Crocodile Lizard? An integrative taxonomic approach reveals Vietnamese and Chinese *Shinisaurus crocodilurus* represent separate conservation and taxonomic units. Der Zoologische Garten. (2016) 85:240–60. 10.1016/j.zoolgart.2016.06.001

[4] Mitchell MA. Mycobacterial infections in reptiles. Vet Clin North Am Exot Anim Pract. (2012) 15:101–11. 10.1016/j.cvex.2011.10.002 22244116

[5] Marschang RE. Viruses infecting reptiles. Viruses. (2011) 3:2087–126. 10.3390/v3112087 22163336 PMC3230843

[6] Mendoza-Roldan JA Ravindran Santhakumari Manoj R Latrofa MS Iatta R Annoscia G Lovreglio P. Role of reptiles and associated arthropods in the epidemiology of rickettsioses: a one health paradigm. PLoS Negl Trop Dis. (2021) 15:e0009090. 10.1371/journal.pntd.0009090 33596200 PMC7888606

[7] Movalli P Krone O Osborn D Pain D. Monitoring contaminants, emerging infectious diseases and environmental change with raptors, and links to human health. Bird Stud. (2018) 65:S96–S109. 10.1080/00063657.2018.1506735

[8] Blanco JL Garcia ME. Animal as Reservoir of Fungal Diseases (Zoonoses?). In: Ahmad I Owais M Shahid M Aqil F, editors. Springer-Verlag Berlin. Heidelberger Platz 3, D-14197 Berlin, Germany (2010). p: 47–70.

[9] Ain-Najwa MY Yasmin AR Omar AR Arshad SS Abu J Mohammed HO. Evidence of West Nile virus infection in migratory and resident wild birds in west coast of peninsular Malaysia. One Health. (2020) 10:100134. 10.1016/j.onehlt.2020.100134 32405525 PMC7210594

[10] Foti M Giacopello C Fisichella V Latella G. Multidrug-resistant *Pseudomonas aeruginosa* isolates from captive reptiles. J Exot Pet Med. (2013) 22:270–4. 10.1053/j.jepm.2013.08.007

[11] Seixas R Pissarra H Santos J Bernardino R Fernandes T Correia J. Severe fibrinonecrotic enteritis caused by *Pseudomonas aeruginosa* in a captive monitor lizard (Varanus niloticus). J Zoo Wildl Med. (2014) 45:410–2. 10.1638/2013-0150R1.1 25000709

[12] Supic J Residbegovic E Koro A Hadziabdic S Golob M Skapur V. Fatal disseminated *Pseudomonas aeruginosa* infection in a captive green iguana (*Iguana iguana*). Acta Vet-Beogr. (2021) 71:361–70. 10.2478/acve-2021-0031

[13] van Schingen M Ihlow F Ziegler T Bonkowski M Wu Z Roedder D. Potential distribution and effectiveness of the protected area network for the crocodile lizard, *Shinisaurus crocodilurus* (Reptilia: Squamata: Sauria). Salamandra. (2014) 50:71–7.

[14] Jiang H Zhang X Li L Ma J He N Liu H. Identification of austwickia chelonae as cause of cutaneous granuloma in endangered crocodile lizards using metataxonomics. PeerJ. (2019) 7:e6574. 10.7717/peerj.6574 30886772 PMC6420803

[15] Tran A Le Minter G Balleydier E Etheves A Laval M Boucher F. Describing fine spatiotemporal dynamics of rat fleas in an insular ecosystem enlightens abiotic drivers of murine typhus incidence in humans. PLoS Negl Trop Dis. (2021) 15:e0009029. 10.1371/journal.pntd.0009029 33600454 PMC7924756

[16] Jiang H Luo S Zhou J Huang W Li L Zhang X. Skin microbiota was altered in crocodile lizards (*Shinisaurus crocodilurus*) with skin ulcer. Front Vet Sci. (2022) 9:817490. 10.3389/fvets.2022.817490 35237680 PMC8884271

[17] Gierloff BC Lefmann G. Pseudomonas aeruginosa. I occurrence in animals and man (author's transl) Nordisk veterinaermedicin. (1974) 26:639–57. 4217443

[18] Roca DAL. *Pseudomonas aeruginosa*: a dangerous adversary. Acta Bioquim Clin Latinoam. (2014) 48:465–74.

[19] Milivojevic D Sumonja N Medic S Pavic A Moric I Vasiljevic B. Biofilm-forming ability and infection potential of Pseudomonas aeruginosa strains isolated from animals and humans. Pathog Dis. (2018) 76:14. 10.1093/femspd/fty041 29684116

[20] Omondi D Masiga DK Fielding BC Kariuki E Ajamma YU Mwamuye MM. Molecular detection of tick-borne pathogen diversities in ticks from livestock and reptiles along the shores and adjacent islands of Lake Victoria and Lake Baringo, Kenya. Front Vet Sci. (2017) 4:73. 10.3389/fvets.2017.00073 28620610 PMC5451513

[21] Xiong Y Wu Q Qin XD Luo SY Yang CS Wu ZJ. Epidemiological survey of skin disease among captive breeding crocodile lizards (*Shinisaurus crocodilurus*). (2021).

[22] Chang WS Li CX Hall J Eden JS Hyndman TH Holmes EC. Meta-transcriptomic discovery of a divergent circovirus and a chaphamaparvovirus in captive reptiles with proliferative respiratory syndrome. Viruses-Basel. (2020) 12:15. 10.3390/v12101073 32992674 PMC7600432

[23] Cota JB Carvalho AC Dias I Reisinho A Bernardo F Oliveira M. Salmonella spp. in pet reptiles in portugal: prevalence and chlorhexidine gluconate antimicrobial efficacy. Antibiotics-Basel. (2021) 10:324. 10.3390/antibiotics10030324 33808891 PMC8003820

[24] Dickinson VM Duck T Schwalbe CR Jarchow JL Trueblood MH. Nasal and cloacal bacteria in free-ranging desert tortoises from the western United States. J Wildl Dis. (2001) 37:252–7. 10.7589/0090-3558-37.2.252 11310875

[25] Doden G Gartlan B Klein K Maddox CW Adamovicz LA Allender MC. Prevalence and antimicrobial resistance patterns of Salmonella spp. in two free-ranging populations of eastern box turtles (Terrapene *Carolina carolina*). J Zoo Wildl Med. (2021) 52:863–71. 10.1638/2020-0061 34687501

[26] Hatt JM. Dermatological diseases in reptiles. Schweiz Arch Tierheilkd. (2010) 152:123–30. 10.1024/0036-7281/a000030 20235013

[27] Sander WE King R Graser W Kapfer JM Engel AI Adamovicz L. Coxiella burnetii in 3 species of turtles in the upper midwest, United States. Emerg Infect Dis. (2021) 27:3199–202. 10.3201/eid2712.211278 34808095 PMC8632166

[28] Tang PK Divers SJ Sanchez S. Antimicrobial susceptibility patterns for aerobic bacteria isolated from reptilian samples submitted to a veterinary diagnostic laboratory: 129 cases (2005-2016). J Am Vet Med Assoc. (2020) 257:305–12. 10.2460/javma.257.3.305 32657653

[29] Parrish K Kirkland PD Skerratt LF Ariel E. Nidoviruses in reptiles: a review. Front Vet Sci. (2021) 8:733404. 10.3389/fvets.2021.733404 34621811 PMC8490724

[30] Brockmann M Aupperle-Lellbach H Muller E Heusinger A Pees M Marschang RE. Aerobic bacteria from skin lesions in reptiles and their antimicrobial susceptibility. Tierarztl Prax Ausg Kleintiere Heimtiere. (2020) 48:78–88. 10.1055/a-1115-7907 32325523

[31] Ebani VV Fratini F Ampola M Rizzo E Cerri D Andreani E. Pseudomonas and Aeromonas isolates from domestic reptiles and study of their antimicrobial *in vitro* sensitivity. Vet Res Commun. (2008) 32 Suppl 1:S195–8. 10.1007/s11259-008-9160-9 18683065

[32] Kubota R Tokiwa T Matsubara K Okamoto M Ike K. Detection and molecular characterization of Cryptosporidium species in wild-caught pet spiny-tailed lizards. Int J Parasitol-Parasit Wildl. (2020) 11:83–7. 10.1016/j.ijppaw.2020.01.002 31956481 PMC6962631

[33] Chen YE Fischbach MA Belkaid Y. Skin microbiota-host interactions. Nature. (2018) 553:427–36. 10.1038/nature25177 29364286 PMC6075667

[34] Knackstedt R Knackstedt T Gatherwright J. The role of topical probiotics in skin conditions: a systematic review of animal and human studies and implications for future therapies. Exp Dermatol. (2020) 29:15–21. 10.1111/exd.14032 31494971

[35] Roudsari MR Karimi R Sohrabvandi S Mortazavian AM. Health effects of probiotics on the skin. Crit Rev Food Sci Nutr. (2015) 55:1219–40. 10.1080/10408398.2012.680078 24364369

[36] Yu Y Dunaway S Champer J Kim J Alikhan A. Changing our microbiome: probiotics in dermatology. Br J Dermatol. (2020) 182:39–46. 10.1111/bjd.18659 31049923

[37] Mottin VHM Suyenaga ES. An approach on the potential use of probiotics in the treatment of skin conditions: acne and atopic dermatitis. Int J Dermatol. (2018) 57:1425–32. 10.1111/ijd.13972 29676446

[38] Chaudhari A Bharti A Dwivedi MK. Chapter 8-Probiotics in the prevention and treatment of atopic skin diseases. In: Dwivedi MK Amaresan N Sankaranarayanan A Kemp EH, editors. Probiotics in the Prevention and Management of Human Diseases. Pittsburgh, PA: Academic Press (2022). p. 117–28. 10.1016/B978-0-12-823733-5.00010-6

[39] Kuebutornye FKA Abarike ED Lu Y. A review on the application of Bacillus as probiotics in aquaculture. Fish Shellfish Immunol. (2019) 87:820–8. 10.1016/j.fsi.2019.02.010 30779995

[40] Redweik GAJ Stromberg ZR Van Goor A Mellata M. Protection against avian pathogenic Escherichia coli and Salmonella Kentucky exhibited in chickens given both probiotics and live Salmonella vaccine. Poult Sci. (2020) 99:752–62. 10.1016/j.psj.2019.10.038 32029160 PMC7587825

[41] Liu C Ponsero AJ Armstrong DG Lipsky BA Hurwitz BL. The dynamic wound microbiome. BMC Med. (2020) 18:358. 10.1186/s12916-020-01820-6 33228639 PMC7685579

[42] Lieke T Meinelt T Hoseinifar SH Pan B Straus DL Steinberg CEW. Sustainable aquaculture requires environmental-friendly treatment strategies for fish diseases. Rev Aquac. (2020) 12:943–65. 10.1111/raq.12365

[43] Talaro KP Chess B. Foundations in Microbiology: McGraw-Hill Science. Hill City, SD: McGraw-Hill Education (2012).

[44] Cheng H-R Jiang N. Extremely rapid extraction of DNA from bacteria and yeasts. Biotechnol Lett. (2006) 28:55–9. 10.1007/s10529-005-4688-z 16369876

[45] Fernandes M Grilo ML Cunha E Carneiro C Tavares L Patino-Martinez J. Antibiotic resistance and virulence profiles of gram-negative bacteria isolated from loggerhead sea turtles (*Caretta caretta*) of the Island of Maio, Cape Verde. Antibiotics-Basel. (2021) 10:12. 10.3390/antibiotics10070771 34202799 PMC8300689

[46] Serra R Grande R Butrico L Rossi A Settimio UF Caroleo B. Chronic wound infections: the role of Pseudomonas aeruginosa and Staphylococcus aureus. Expert Rev Anti-Infect Ther. (2015) 13:605–13. 10.1586/14787210.2015.1023291 25746414

[47] Hanna N Sun P Sun Q Li XW Yang XW Ji X. Presence of antibiotic residues in various environmental compartments of Shandong province in eastern China: its potential for resistance development and ecological and human risk. Environ Int. (2018) 114:131–42. 10.1016/j.envint.2018.02.003 29501851

[48] Wareth G El-Diasty M Abdel-Hamid NH Holzer K Hamdy MER Moustafa S. Molecular characterization and antimicrobial susceptibility testing of clinical and non-clinical Brucella melitensis and Brucella abortus isolates from Egypt. One Health. (2021) 13:100255. 10.1016/j.onehlt.2021.100255 34027005 PMC8122161

[49] Zhai KF Duan H Cui CY Cao YY Si JL Yang HJ. Liquiritin from *Glycyrrhiza uralensis* attenuating rheumatoid arthritis *via* reducing inflammation, suppressing angiogenesis, and inhibiting MAPK signaling pathway. J Agric Food Chem. (2019) 67:2856–64. 10.1021/acs.jafc.9b00185 30785275

[50] Monteagudo-Mera A Rastall RA Gibson GR Charalampopoulos D Chatzifragkou A. Adhesion mechanisms mediated by probiotics and prebiotics and their potential impact on human health. Appl Microbiol Biotechnol. (2019) 103:6463–72. 10.1007/s00253-019-09978-7 31267231 PMC6667406

[51] Kogut MH. The effect of microbiome modulation on the intestinal health of poultry. Anim Feed Sci Technol. (2019) 250:32–40. 10.1016/j.anifeedsci.2018.10.008

[52] Wu XX Teame T Hao Q Ding QW Liu HL Ran C. Use of a paraprobiotic and postbiotic feed supplement [HWF (TM)] improves the growth performance, composition and function of gut microbiota in hybrid sturgeon (*Acipenser baerii* x *Acipenser schrenckii*). Fish Shellfish Immunol. (2020) 104:36–45. 10.1016/j.fsi.2020.05.054 32473360

[53] Xia Y Wang M Gao FY Lu MX Chen G. Effects of dietary probiotic supplementation on the growth, gut health and disease resistance of juvenile Nile tilapia (*Oreochromis niloticus*). Anim Nutr. (2020) 6:69–79. 10.1016/j.aninu.2019.07.002 32211531 PMC7082692

[54] Alhubail A Sewify M Messenger G Masoetsa R Hussain I Nair S. Microbiological profile of diabetic foot ulcers in Kuwait. PLoS ONE. (2020) 15:e0244306. 10.1371/journal.pone.0244306 33378365 PMC7773204

[55] Sala A Di Iannii F Pelizzone I Bertocchi M Santospirito D Rogato F. The prevalence of *Pseudomonas aeruginosa* and multidrug resistant *Pseudomonas aeruginosa* in healthy captive ophidian. Peer J. (2019) 7:13. 10.7717/peerj.6706 30997288 PMC6463849

[56] Plowright RK Parrish CR McCallum H Hudson PJ Ko AI Graham AL. Pathways to zoonotic spillover. Nat Rev Microbiol. (2017) 15:502–10. 10.1038/nrmicro.2017.45 28555073 PMC5791534

[57] Tourkantonis A Heinze V. Carbencillin in *Pseudomonas aeruginosa* infections. Deutsche medizinische Wochenschrift. (1946) 94:914–5. 10.1055/s-0028-1111141 4976102

[58] Poole K. *Pseudomonas aeruginosa*: resistance to the max. Front Microbiol. (2011) 2:13. 10.3389/fmicb.2011.00065 21747788 PMC3128976

[59] Bowler PG Duerden BI Armstrong DG. Wound microbiology and associated approaches to wound management. Clin Microbiol Rev. (2001) 14:244–69. 10.1128/CMR.14.2.244-269.2001 11292638 PMC88973

[60] Makarov VV Sukharev OI Kolomytsev AA. Veterinary epidemiology of propagated infections. Vestnik Rossiiskoi akademii sel'skokhozyaistvennykh nauk. (2009) 70–2.

