# Supplementary figures and images for: Identification of Pseudomonas aeruginosa From the Skin Ulcer Disease of Crocodile Lizards (Shinisaurus crocodilurus) and Probiotics as the Control Measure
Source: Front Vet Sci. 2022 Apr 21;9:850684. doi: 10.3389/fvets.2022.850684 (PMC9069141; doi:10.3389/fvets.2022.850684)

a

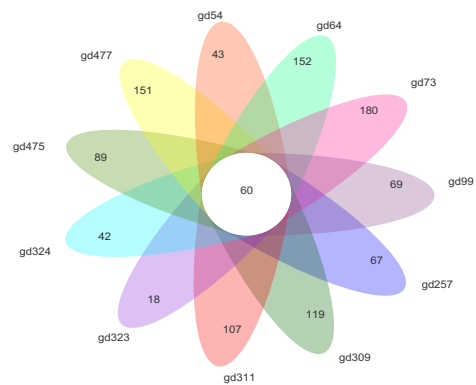

Community barplot analysis

c

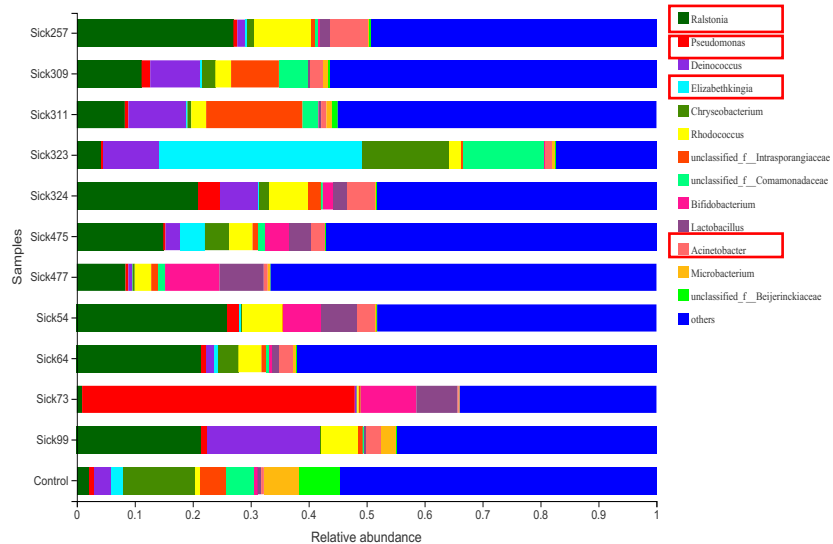

b

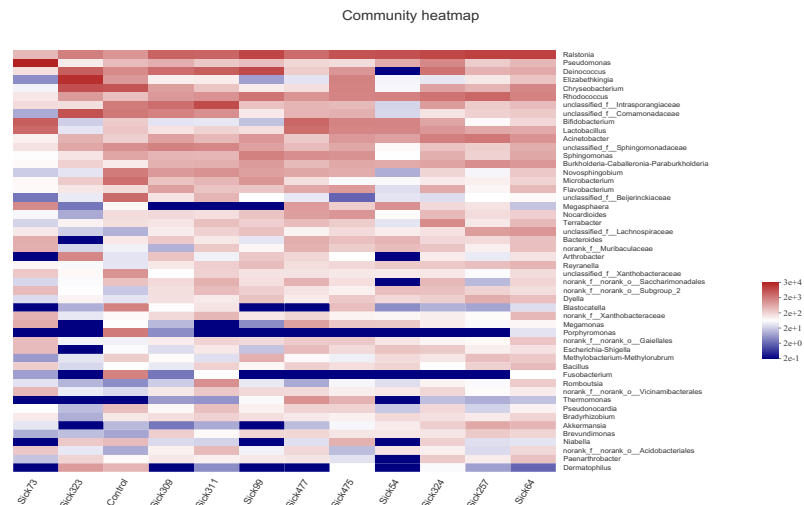

Supplement: Supplementary file 2 [file Image_1.PDF]

Rarefaction curves

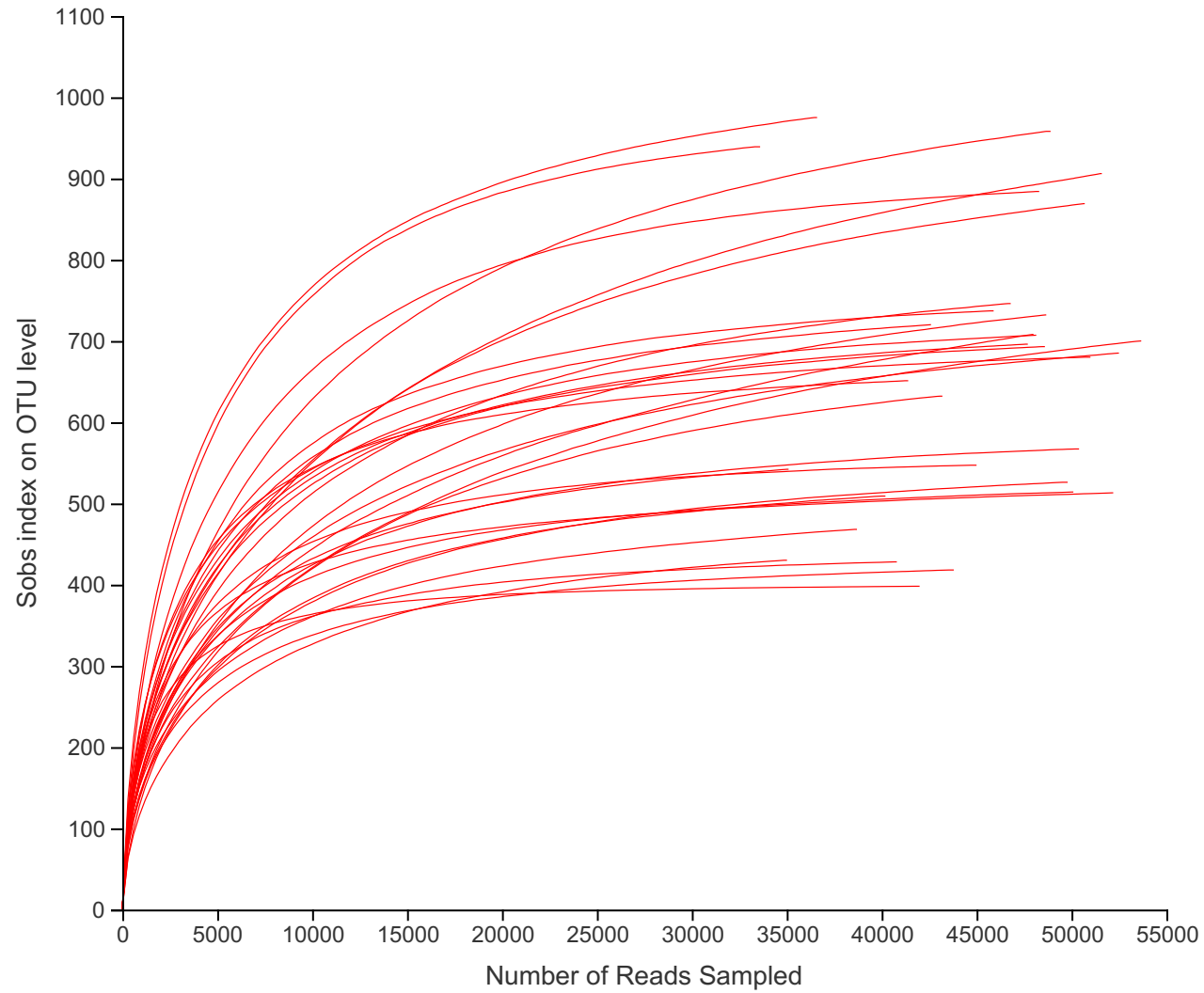

Supplement: Supplementary file 3 [file Image_2.PDF]

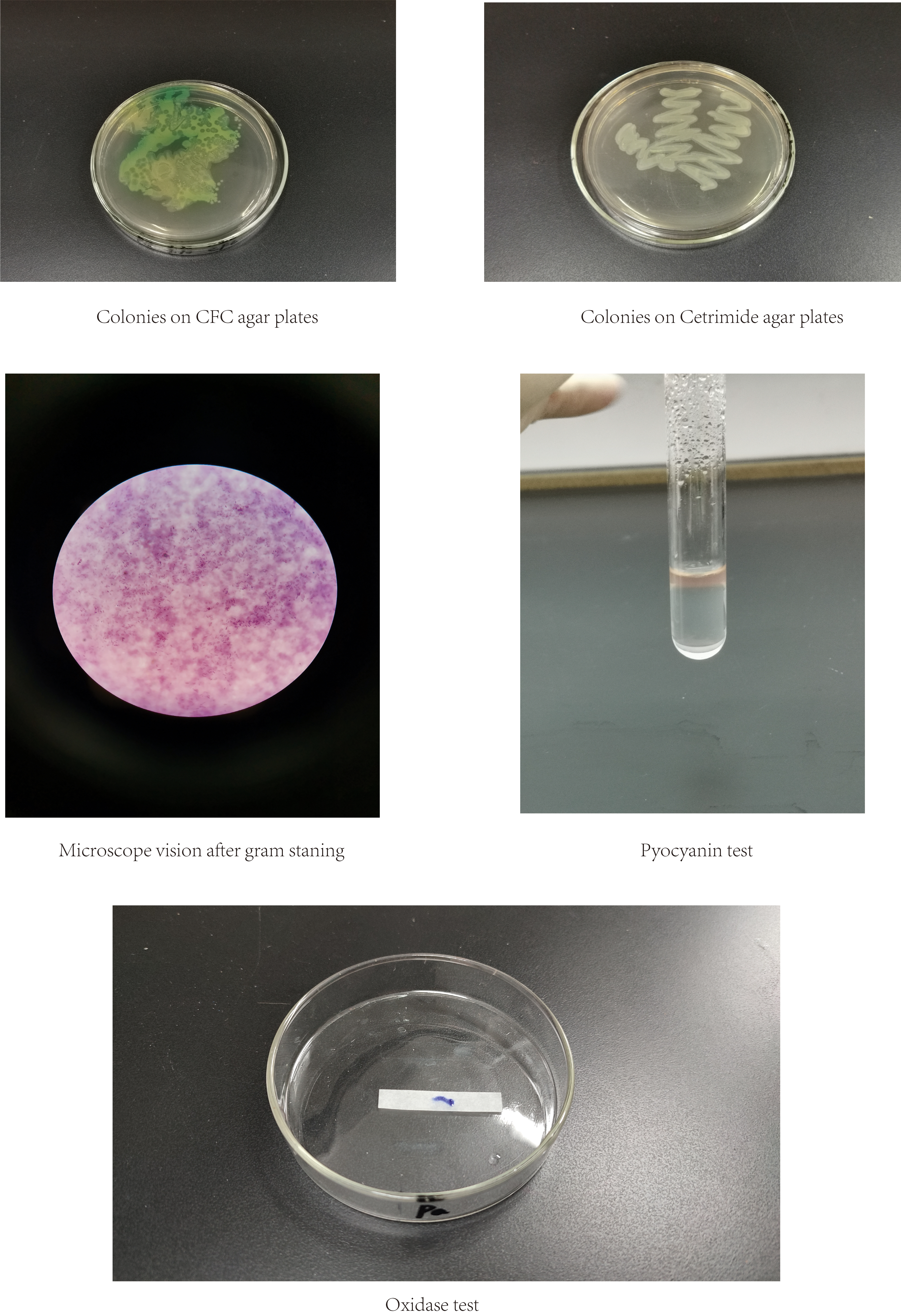

Supplement: Supplementary file 4 [file Image_3.JPEG]
